# Self-Assembled Chitosan/Dialdehyde Carboxymethyl Cellulose Hydrogels: Preparation and Application in the Removal of Complex Fungicide Formulations from Aqueous Media

**DOI:** 10.3390/polym15173496

**Published:** 2023-08-22

**Authors:** Claudiu-Augustin Ghiorghita, Maria Marinela Lazar, Luminita Ghimici, Maria Valentina Dinu

**Affiliations:** “Petru Poni” Institute of Macromolecular Chemistry, Grigore Ghica Voda Alley 41 A, 700487 Iasi, Romania; claudiu.ghiorghita@icmpp.ro (C.-A.G.); maria.lazar@icmpp.ro (M.M.L.); lghimici@icmpp.ro (L.G.)

**Keywords:** hydrogels, chitosan, dialdehyde carboxymethyl cellulose, sorption, fungicides

## Abstract

Environmental contamination with pesticides occurs at a global scale as a result of prolonged usage and, therefore, their removal by low-cost and environmentally friendly systems is actively demanded. In this context, our study was directed to investigate the feasibility of using some self-assembled hydrogels, comprising chitosan (CS) and carboxymethylcellulose (CMC) or dialdehyde (DA)-CMC, for the removal of four complex fungicide formulations, namely Melody Compact (MC), Dithane (Dt), Curzate Manox (CM), and Cabrio^®^Top (CT). Porous CS/CMC and CS/DA-CMC hydrogels were prepared as discs by combining the semi-dissolution acidification sol-gel transition method with a freeze-drying approach. The obtained CS/CMC and CS/DA-CMC hydrogels were characterized by gel fraction yield, FTIR, SEM, swelling kinetics, and uniaxial compression tests. The batch-sorption studies indicated that the fungicides’ removal efficiency (RE%) by the CS/CMC hydrogels was increased significantly with increasing sorbent doses reaching 94%, 93%, 66% and 48% for MC, Dt, CM and CT, respectively, at 0.2 g sorbent dose. The *RE* values were higher for the hydrogels prepared using DA-CMC than for those prepared using non-oxidized CMC when initial fungicide concentrations of 300 mg/L or 400 mg/L were used. Our results indicated that CS/DA-CMC hydrogels could be promising biosorbents for mitigating pesticide contamination of aqueous environments.

## 1. Introduction

Over time, pesticides have become a significant component of modern farming practices and have brought many benefits. Pesticides are mainly used to control or manage pests that can damage crops and reduce agricultural productivity. These pests include insects, snails and rodents, as well as pathogens such as fungi and bacteria that can lead to plant diseases [1,2,3]. However, some pesticides have a negative impact on the environment because they persist in soil or end up in water sources, affecting plants, animals and aquatic ecosystems [1,2,3]. Moreover, constant use of the same pesticides can lead to the development of pest resistance. Furthermore, pesticides can have adverse effects on human health, particularly when used inappropriately or with prolonged exposure [1,2,3]. All these negative effects have challenged the scientific world to find solutions to control and detect their presence in groundwater, surface water sources, and drinking water and, thus, to manage as much as possible the safety and sustainability of pesticide use [4,5,6,7]. Consequently, several analytical methods have been developed and applied to monitor pesticide residues in food and environmental samples, including electrochemical techniques [8] and liquid and gas chromatography coupled with mass spectrometry [9,10]. Biological treatments, precipitation, flocculation, and advanced oxidation processes have also been employed to remove pesticides from aqueous media [11,12,13]. Nevertheless, some pesticides are found in complex formulations with various concentrations, meaning that the above-mentioned approaches are not applicable. Thus, the use of adsorbent-based techniques such as stir bar sorptive extraction [14], solid-phase micro-extraction [14,15], and magnetic solid-phase extraction [16,17] have gained growing attention in recent years. These adsorption techniques are particularly suitable because of their simplicity, ‘green’ nature, as they are conducted in water, and the availability of a wide range of cheap sorbents [5,6,7,18,19,20,21,22,23,24]. For instance, natural or modified clays [5,7,11,25], activated carbon [11,26], and zeolites [11,26] have been reported as efficient sorbents for pesticide removal from aqueous solutions. Even though the sorbents mentioned above are low cost and environmentally friendly, their recovery and regeneration after pesticide removal is a difficult task. The use of biopolymers as support matrices for zeolites allows for the formation of new composite materials that overcome the aforementioned shortcoming [27]. In addition, the great attention given to biopolymers derives also from their intrinsic features, such as biodegradability, non-toxicity, biocompatibility and bioactivity. These properties, combined with the advantage of having many functional groups on its backbone (-OH and NH_2_), have made chitosan (CS) particularly interesting for biomedical applications [28,29], as well as for the removal of heavy metal ions, pesticides, dyes, and oxyanions, in soluble form [30,31] or in composite systems as micro/macro-hydrogel 3D networks [32,33,34,35,36,37].

Apart from these advantages, CS is also recognized for its low stability in acidic environments and weak mechanical properties [38]. Thus, by blending or grafting CS with other natural or synthetic polymers, biocomposite materials with superior properties and sorption performances have been obtained [38,39,40]. In this context, our study aims to put together the benefits of CS, as a polycation component, and of a polyanion, carboxymethylcellulose (CMC), to design novel polyelectrolyte complex (PEC) hydrogels with high sorption performances for pesticides. It is well known that the synthesis of PECs is a versatile approach to obtain novel materials by spontaneous electrostatic interactions between oppositely charged polyions [41,42,43,44,45,46,47,48].

To be suitable for wastewater purification, PEC-based adsorbents should possess several key characteristics:

(i) Macroporosity: The adsorbents need to have a porous structure with interconnected channels or void spaces that allow water to pass through while retaining contaminants, thus ensuring a high flow rate and preventing clogging.
(ii) Mechanical stability: Adsorbents should be able to withstand compression forces without losing their filtration efficiency. This feature is important for maintenance, cleaning, and reusability purposes.
(iii) Reusability: To minimize waste and cost, it is desirable for PEC adsorbents to be reusable through multiple sorption/desorption cycles without significant loss in their performance. This implies that the adsorbents should maintain their adsorption capacity and structural integrity over time.

To achieve these desired characteristics, several factors need to be considered during the engineering process of PECs, such as: (1) the molar mass and charge density of polyions; (2) the mixing mode of polyions; (3) the hydrophilic/hydrophobic balance of the oppositely charged polyions; and (4) the ionic strength of the environment [41,42,43,44,45,46,47,48]. Obviously, fabrication of efficient PEC-based adsorbents for wastewater treatment applications is still a challenge.

The strategy adopted in this work to design porous PEC hydrogels involves combining the semi-dissolution acidification sol-gel transition method with a freeze-drying approach. The CS/CMC hydrogels were stabilized mainly by the electrostatic interactions between the oppositely charged groups of the two components. Chemically stabilized PEC hydrogels were also designed by employing selectively oxidized CMC (DA-CMC) polymers with free aldehyde groups in their structure. The aldehyde content of DA-CMC polymers was determined by the hydroxylamine method, while the content of carboxyl groups of pristine and oxidized CMC was determined by conductometric titration. The transformation of reactants in hydrogels was evaluated by determining the gel fraction yield, whereas the structural and morphological characterization was achieved by FTIR and SEM. The mechanical properties and the stability of the matrices at various pH values were also evaluated. The sorption investigation of the PEC hydrogels was performed in batch-mode using four commercial fungicide formulations, namely Melody Compact (MC), Dithane (Dt), Curzate Manox (CM), and Cabrio^®^Top (CT). The influence of the sorbent dose and initial suspension concentration on the sorption capacity of the CS/CMC and CS/DA-CMC hydrogels were investigated.

## 2. Materials and Methods

### 2.1. Materials

**Figure 1:**
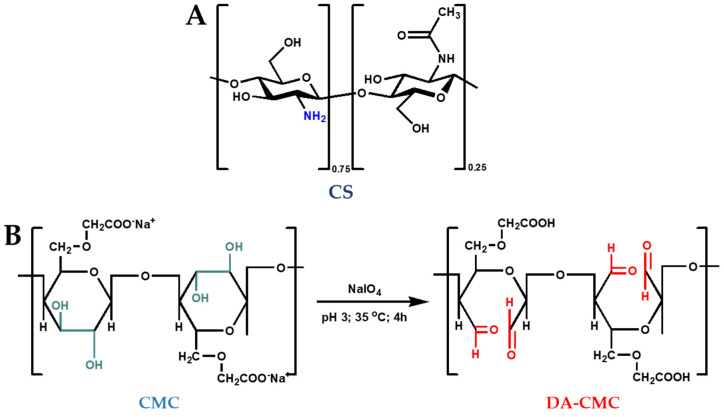
The structure of CS (**A**) and the oxidation of CMC to DA-CMC using NaIO_4_ (**B**).

CS with molar mass of 236 kDa and deacetylation degree (DD) of about 75%, carboxymethyl cellulose sodium salt (CMC) with molar mass and substitution degree of 90 kDa and 0.7 (denoted as CMC1), or 250 kDa and 0.9 (denoted as CMC2) (Figure 1), and sodium periodate (NaIO_4_) were purchased from Sigma-Aldrich (Steinheim, Germany), and were used as received. Dithane M45 (Dow Agro Sciences B.V., Indianapolis, IN, USA) as wettable powder, Melody Compact 49 WG (Bayer AG, Leverkusen, Germany) as water-dispersible granules, Cabrio^®^Top (BASF, Basel, Switzerland) as powder, and Curzate Manox (Du Pont de Nemours, Wilmington, DE, USA) were purchased from a local store. H_2_SO_4_, acetic acid, HCl, NaOH and hydroxylamine hydrochloride were purchased from Chemical Company (Iași, Romania). Throughout the experiments, all solutions and suspensions were prepared using distilled water.

### 2.2. Oxidation of CMC

CMC with two molar masses (CMC1 = 90 kDa and CMC2 = 250 kDa) was oxidized by reaction with NaIO_4_ (Figure 1B) [49,50]. Briefly, CMC (5.0 g) was first dissolved in 250 mL water, and then the pH of the solutions was adjusted to 3 using 1 M H_2_SO_4_. Afterwards, they were transferred to a 500 mL round bottom flask, and precise amounts of NaIO_4_ were added to obtain the desired molar ratios. The oxidation reaction was conducted in the dark, at 35 °C, for 4 h. The obtained solutions were dialyzed for 4 days against distilled water; the dried dialdehyde-CMC (DA-CMC) polymers were obtained by lyophilization.

### 2.3. Hydrogels Preparation

Self-assembled hydrogels comprising CS and CMC or DA-CMC, were prepared as discs using the semi-dissolution acidification sol-gel transition method [51], with some modifications. First, CMC and DA-CMC aqueous solutions were prepared at 1 wt.% and 2 wt.% concentrations, their pH being adjusted afterwards to 7.2 with 0.1 M HCl or 1 M NaOH. Certain volumes of CMC or DA-CMC solutions were added to circular weighing bottles, which were then cooled in an ice-bath. Subsequently, a certain amount of CS powder was added to ensure a pre-established weight ratio. Table 1 lists the preparation conditions of the different PEC sponges.

The dispersions were kept under strong magnetic stirring (1200 rpm) for 3 min, afterwards being placed for 24 h in a desiccator containing acetic acid. After this time, the obtained gels were thoroughly washed with water to remove the acetic acid residues or the unreacted polymer chains, frozen in liquid nitrogen and freeze-dried in a Martin Christ, ALPHA 1-2LD device (Kansas City, MO, Fort Scott, KS, USA) (48 h, −57 °C, 0.045 mbar).

### 2.4. Characterization Methods

#### 2.4.1. Aldehyde Content

The aldehyde content (*AC*, %) of DA-CMC polymers was determined by the hydroxylamine method, which involves the conversion of aldehyde groups to oximes by Schiff base formation, with the release of hydrochloric acid [49,52]. The experimental procedure was as follows: 0.1 g of DA-CMC samples was dissolved in 25 mL water, and the solution pH was adjusted to 5 using 1 M NaOH solution. Subsequently, 20 mL of 10 mg/mL hydroxylamine hydrochloride at pH 5 was added, and the reaction was conducted for 5 h at 40 °C under magnetic stirring. The formed HCl was titrated with 0.1 M NaOH until the pH of the solution increased to 5. For control, the same procedure was applied to the non-oxidized CMC. The *AC* has been calculated with Equation (1):

(1) AC, %=CNaOH(Vc−Vb)(1000×mMus)×100

where *C_NaOH_* is the concentration of NaOH (mol/L) used for titration, *m* is the dry weight (g) of the DA-CMC sample, *M_us_* is the molar mass (g/mol) of the DA-CMC repeating unit, and *V_c_* and *V_b_* are the volumes (mL) of NaOH consumed to titrate the DA-CMC and non-oxidized CMC samples, respectively. The experiments were performed in duplicate.

#### 2.4.2. Carboxyl Groups Content

The content of carboxyl groups of pristine and oxidized CMC was determined by conductometric titration, according to a slightly modified literature protocol [49,53]. Briefly, 50 mg of CMC or DA-CMC and 5 mL of 10^−2^ M NaCl were added to 95 mL of water and stirred until complete dissolution. Next, the solution’s pH was adjusted to ≈ 2.8 with 0.1 M HCl. The conductometric titration was performed by adding 0.1 M NaOH at a speed of 0.1 mL/min. The recorded conductivity values were represented as a function of NaOH volume consumed, the carboxyl groups’ content being determined from the volume of NaOH consumed in the weak acid curve section in the graphs using Equation (2) [49].

(2) $$C_{COOH}=\frac{V_{NaOH}\times {\;C}_{NaOH}}{1000\times \;m},\;mmol/g$$

where *C_COOH_* (mmol/g) is the content of carboxyl groups in the CMC or DA-CMC samples, *V_NaOH_* (mL) and *C_NaOH_* (mol/L) are the volume and concentration, respectively, of NaOH used for titration, and *m* (g) is the dry weight of CMC or DA-CMC. The experiments were performed in duplicate.

#### 2.4.3. Structural Characterization

FTIR spectra of CMC, DA-CMC and of the hydrogels were recorded by a Bruker Vertex FT-IR spectrometer, at a resolution of 2 cm^−1^, in the range 4000–400 cm^−1^, by KBr pellet technique.

^1^H NMR spectra of non-oxidized and oxidized CMC were recorded on a Bruker Avance NEO 400 MHz spectrometer (Bruker BioSpin, Rheinstetten, Germany), operating at 400.1 MHz. The spectra were recorded at 24 °C in deuterium oxide (D_2_O). Chemical shifts are reported in δ units (ppm) and are referenced to sodium 3-(trimethylsilyl)-[2,2,3,3-d4]-1-propionate internal standard at 0.0 ppm.

#### 2.4.4. Gel Fraction Yield

The transformation of reactants in hydrogels was evaluated by determining the gel fraction yield (*GFY*, %), using Equation (3):

(3) $$GFY,\;\%=\frac{W_{d}}{W_{0}}\times 100$$

where *W_d_* (g) is the weight of dried hydrogels (under vacuum in the presence of P_2_O_5_ until constant weight) and *W_0_* (g) is the weight of used reactants [54].

#### 2.4.5. Morphology of Sponges

Scanning Electron Microscopy (SEM) images of the hydrogels were recorded with a Verios G4 UC (Thermo Scientific, Brno, Czech Republic) device equipped with an energy dispersive X-ray (EDX) analyzer (Octane Elect Super SDD detector, Mahwah, NJ, USA). Prior to analysis, the sponges were coated with a 10 nm platinum layer using a Leica EM ACE200 Sputter. The SEM analysis was performed in high vacuum mode, using a concentric backscattered or an Everhart-Thornley detector, and at an accelerating voltage of 10 kV. The pore sizes of CS/CMC or CS/DA-CMC hydrogels were evaluated from SEM micrographs using Image J 1.53 v software by averaging at least 15 measured pores per sample. The porosity (*P*, %) of the hydrogels was determined using Equation (4) [54,55,56]:

(4) P=1−ρappρs×100

where *ρ_app_* and *ρ_s_* are the apparent density of prepared hydrogels and the specific density of polymeric skeleton (*ρ_s_ =* 1.4063 g/cm^3^), respectively. *ρ_app_* (g/cm^3^) was calculated using Equation (5) [54,55]:

(5) $$\rho_{app}=\frac{m}{V}$$

where *m* and *V* are the weight and volume, respectively, of rectangular shaped samples.

#### 2.4.6. Swelling Properties

The swelling kinetics of hydrogels were evaluated by immersing about 10 mg of dry samples in water. At incremental time intervals, the swollen samples were removed from water and weighed, after wiping away the excess of water with filter paper. The *SR* was calculated by Equation (6):

(6) $$SR=\frac{W_{t}}{W_{d}}$$

where *W_d_* (g) is the weight of dried samples, and *W_t_* (g) is the weight of swollen samples at time *t*. The measurements were performed in duplicate, the results being expressed as average values ± standard deviation.

#### 2.4.7. Mechanical Tests

Mechanical tests were performed on equilibrium-swollen hydrogels by applying a compression force of 100 N at a cross-head speed of 1 mm/min. The elastic modulus, the compressive strength, and the maximum compression strain were evaluated according to the protocols already published for other PEC-based hydrogels [47,48].

#### 2.4.8. Erosion Study

The stability of hydrogels in aqueous media with different pHs was investigated according to a recently published protocol, slightly modified [47]. Hydrogel samples weighing about 25 mg were introduced in 20 mL aqueous solution with pH 2, pH 6 or pH 10, and stirred for 3 h at 150 rpm in an ES-20 Orbital Shaker-Incubator. Then, the samples were separated, freeze-dried, and weighed again. The hydrogels stability was calculated with Equation (7):

(7) $$Stability,\;\%=\frac{W_{t}}{W_{0}}\times 100$$

where *W_0_* (g) and *W_t_* (g) are the weights of the initial hydrogel samples, and after erosion study.

### 2.5. Sorption of Fungicide Formulations

**Figure 2:**
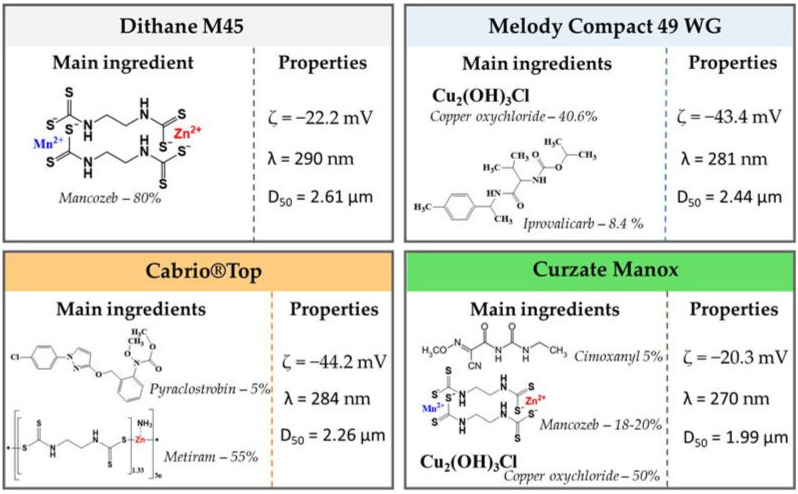
The composition and main physico-chemical properties of the commercial fungicides.

The sorption of four commercial fungicide formulations (Dithane M45 (Dt), Melody Compact 49 WG (MC), Curzate Manox (CM) and Cabrio^®^Top (CT)) by the PEC hydrogels was performed in batch mode, at room temperature. The main constituents of the fungicide formulations and some properties of their suspensions are displayed in Figure 2 [12].

The fungicide sorption performance by the PEC hydrogels was evaluated with respect to sorbent dose and initial suspension concentration. A known volume of fungicide suspension at fixed initial concentration (*C_0_* = 200 mg/L) was mixed with an amount of sorbent, under constant stirring speed (200 rpm) for 24 h. Afterwards, the samples were collected and filtered; the absorbance of the residual fungicide suspension in the filtrate was measured with a SPECOL 1300 Analytic Jena spectrophotometer at corresponding wavelength (λ, Figure 2).

The sorption data were expressed in terms of removal efficiency (*RE*, %), that has been calculated by Equation (8):

(8) $$RE,\;\%=\frac{\left(C_{0}-C_{e}\right)}{C_{0}}\times 100$$

where *C*_0_ (mg/L) and *C_e_* (mg/L) are the initial and equilibrium concentrations of fungicide suspensions.

## 3. Results and Discussion

### 3.1. Oxidation of CMC

The oxidation of CMC by NaIO_4_ in aqueous medium at acidic pH leads to the selective oxidation of hydroxyl groups at the C_2_ and C_3_ positions of anhydroglucose units (AGU), with the formation of a dialdehyde structure (Figure 1). The efficiency of this reaction is influenced by several operational parameters, including the temperature, the pH, and the AGU:NaIO_4_ molar ratio [49]. Herein, we investigated the influence of CMC molar mass and AGU:NaIO_4_ molar ratio on the oxidation reaction efficiency (Table 2). The oxidation degree of CMC was evaluated as a function of the *AC* values, which have been determined by the hydroxylamine method (Section 2.4.1).

As seen in Table 2, a slightly higher oxidation degree was found for CMC1 (*AC* = 68.9%) than for CMC2 (*AC* = 60.2%) at equimolar AGU:NaIO_4_ ratio, which could be ascribed to the lower viscosity of the former polymer solution. Furthermore, in the case of CMC2 oxidation, a non-linear dependence between NaIO_4_ dosage and *AC* was found (23.8%, 40.7% and 60.2% for the DA-CMC2.1, DA-CMC2.2 and DA-CMC2.3, respectively). At the same time, the increase in NaIO_4_ dosage led to a slight increase in *C_COOH_* (Table 2), as determined by conductometric titration (Figure S1). This indicates the possible degradation of CMC at high NaIO_4_ dosages, with the formation of additional carboxyl groups [52].

**Figure 3:**
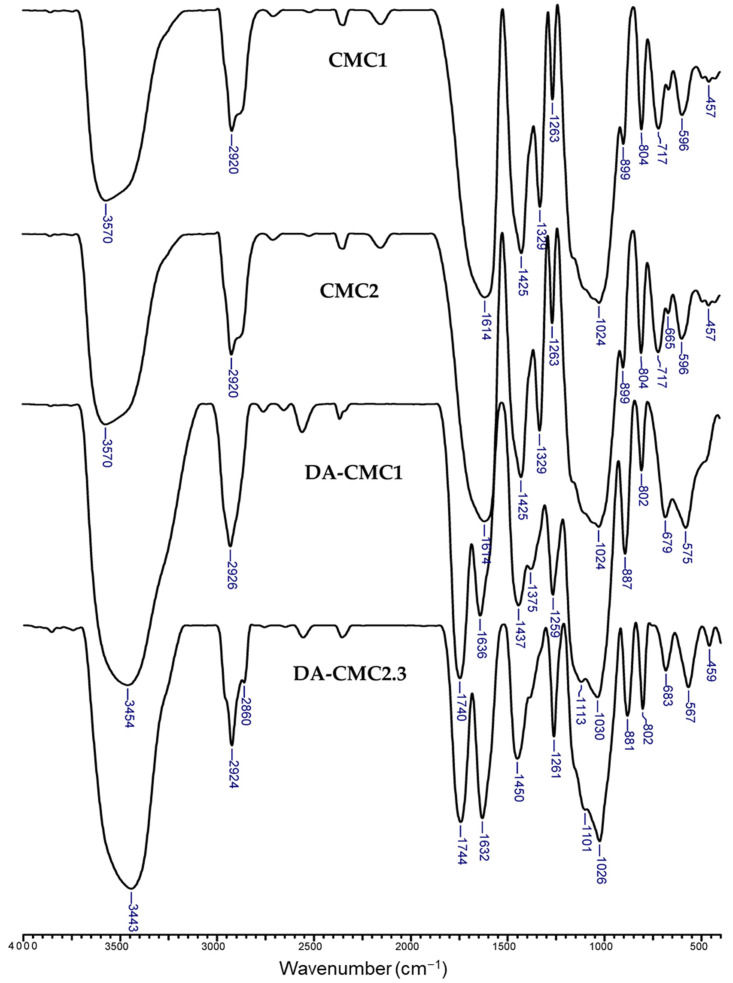
FTIR spectra of CMC1 and CMC2 before and after oxidation at 1:1 AGU:NaIO_4_ molar ratio.

Figure 3 shows the FTIR spectra of CMC1 and CMC2 before and after oxidation at 1:1 AGU:NaIO_4_ molar ratio.

The FTIR spectra of DA-CMC2.2 and DA-CMC2.3 samples are presented in Figure S2. The main characteristic bands of pristine CMC are: 3570 cm^−1^, attributed to O-H stretching vibrations and to intra- and/or intermolecular hydrogen bonds; 2920 cm^−1^, corresponding to C-H stretching vibrations; 1614 cm^−1^ and 1425 cm^−1^, assigned to asymmetric and symmetric vibrations, of COO^−^ groups; 1329 cm^−1^ and 1263 cm^−1^, assigned to symmetric stretching of COO^−^and -OH bending vibrations, respectively; and 1024 cm^−1^, which has been attributed to the skeletal vibrations of AGU in the CMC structure [48,57,58]. The oxidation of CMC1 and CMC2 (Figure 3 and Figure S2) led to the following spectral changes:

(i) The appearance of new bands at 1738–1744 cm^−1^ and at 881–893 cm^−1^ in the spectra of DA-CMC1, DA-CMC2.2 and DA-CMC2.3, characteristic to the vibrations of aldehyde C=O groups, and to the formation of hemiacetals between aldehyde groups and vicinal hydroxyl groups, respectively [57,59,60,61].
(ii) The disappearance of the bands at 1329 cm^−1^, which was associated with the protonation of carboxylate groups (-COO^−^ → -COOH) [62];
(iii) Blue-shift of the band at 3570 cm^−1^ to 3441–3456 cm^−1^, which could be correlated with the decrease in hydroxyl group content following the oxidation reaction [57].
(iv) Red-shift of the band at 1614 cm^−1^ to 1636 cm^−1^; 1637 cm^−1^; and 1632 cm^−1^, for DA-CMC1, DA-CMC2.2 and DA-CMC2.3, respectively, that are assigned to conjugated carboxylate groups [57].
(v) Red-shift of the bands at 1425 cm^−1^ to 1437 cm^−1^; 1431 cm^−1^; and 1450 cm^−1^, in the spectra of DA-CMC1, DA-CMC2.2 and DA-CMC2.3, respectively, that were also attributed to structural changes in carboxylate C=O groups.

These spectral changes were not entirely visible in the spectrum of DA-CMC2.1 (Figure S2), probably because of its lower *AC*.

**Figure 4:**
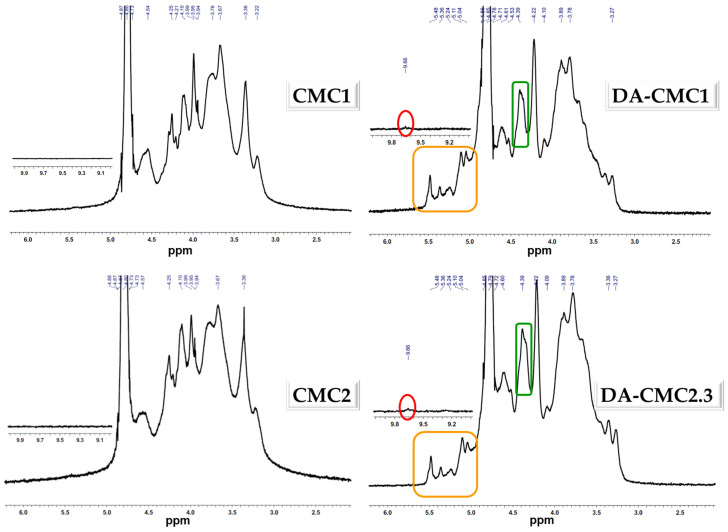
^1^H NMR spectra of CMC1 and CMC2 before and after oxidation at 1:1 AGU:NaIO_4_ molar ratio.

The oxidation of CMC was also investigated by ^1^H NMR (Figure 4).

A plethora of ^1^H resonances are seen in the region of 4.57–3.22 ppm of CMC1 and CMC2, that arise from the non- or carboxymethyl-substituted AGUs [63]. Considering that the substitution degrees of CMC1 and CMC2 were 0.7 and 0.9, respectively, it is expected that most AGUs are mono-carboxymethylated, a small fraction of them being non-substituted. Significant spectral changes were noted for both DA-CMCs. For example, the shift of ^1^H signals at 4.25 ppm to 4.39 ppm, as well as the appearance of new ^1^H resonances in the region of 5.58–5.04 ppm, are related to CH and OH protons whose electron densities were influenced by the formation of aldehyde groups. Furthermore, the aldehyde protons were also evidenced by a small singlet signal at 9.66 ppm [57].

### 3.2. Preparation and Characterization of Hydrogels

CS/CMC and CS/DA-CMC hydrogels with different compositions (Table 1) have been prepared by the self-assembly of polycation/polyanion pairs using the delayed acidification of a dispersion of CS in polyanion solutions under a gaseous acetic acid atmosphere. Under such an atmosphere, the amino groups of CS are gradually protonated (-NH_2_ → NH_3_^+^), thus promoting the solubilization of CS in the polyanion solutions [50,64]. In the case of CS/CMC PECs, the gel’s stabilization is achieved mainly by the electrostatic interactions between the oppositely charged groups of the two components, hydrogen bonds also being possible [46,48,64]. However, because the oxidized CMCs contain free aldehyde groups in their structure, the stabilization of CS/DA-CMC hydrogels implies also the formation of covalent imine bonds with the amino groups of CS. An early proof of this was provided by the optical images of the two types of PEC gels immediately after removal from the acetic acid atmosphere. As depicted in Figure S3, the CS/CMC2 gels were white, while the CS/DA-CMC2.3 ones were light brown, a color characteristic to gels stabilized by imine bonds [54,65].

Insight into the density of interpolymeric interactions in the prepared hydrogels was obtained by calculating the NH_2_/COOH and NH_2_/C=O molar ratios (Table S1), according to Equation (9) and Equation (10), respectively.

(9) $$\frac{{mmol}_{NH2}}{{mmol}_{COOH}}=\frac{\frac{m_{CS}\times 1000}{M_{us,CS}}\times DD}{C_{COOH}\times m_{CMC}}$$

(10) $$\frac{{mmol}_{NH2}}{{mmol}_{CO,\;DA-CMC}}=\frac{\frac{m_{CS}\times 1000}{M_{us,CS}}\times DD}{\frac{m_{DA-CMC}\times 1000}{M_{us,DA-CMC}}\times AC}$$

where: *m_CS_*, *m_CMC_* and *m_DA-CMC_* are the weight of CS, CMC and DA-CMC, respectively, that were used to prepare the hydrogels (Table 1); *M_us,CS_* and *M_us,DA-CMC_* are the average molar masses of the CS and DA-CMC structural units, respectively; DD is the deacetylation degree of CS; AC is the aldehyde content of DA-CMC samples (Table 2); and *C_COOH_* is the concentration of carboxyl groups (Table 2).

The NH_2_/COOH molar ratio gives information on the proportion of functional groups in the two polymer components that are available to form electrostatic interactions. The closer this value is to parity, the better the stoichiometry of functional groups. Values higher than one correspond to an excess of NH_2_ groups, while lower values indicate an excess of COOH groups. The proportion of NH_2_ and COOH groups in the prepared hydrogels was clearly influenced by the weight ratio of the used polymers, but also, to some extent, by the content of the COOH groups in CMC or DA-CMC. As expected, the only hydrogels with an NH_2_/COOH molar ratio lower than one were those prepared at 0.5/1 weight ratio of CS/CMC or CS/DA-CMC. On the contrary, the highest NH_2_/COOH molar ratios were found for the hydrogels prepared at 1.5/1 weight ratio of CS/CMC or CS/DA-CMC. In the case of the hydrogels prepared at 1/1 weight ratio of CS/CMC or CS/DA-CMC, the slight variation of the NH_2_/COOH molar ratio was influenced by the content of the COOH groups in the polyanions, decreasing with the increase in *C_COOH_* (Table 2).

In addition to electrostatic interactions, the hydrogels prepared with DA-CMC could also be stabilized by imine bonds. In this respect, it has been found that the calculated NH_2_/C=O molar ratios were influenced by the *AC* (%) of DA-CMC samples and by the hydrogels’ composition (Table S1). An NH_2_/C=O molar ratio of 1.29 was calculated for the CS/DA-CMC2.3 hydrogels prepared at 0.5/1 weight ratio, indicating an excess of carbonyl groups in the system, while for all the other compositions the NH_2_/C=O molar ratios were lower that one, which corresponds to an excess of amino groups. However, the formation of covalent imine bonds precedes the generation of electrostatic interactions, which results in the fabrication of brittle hydrogels.

The PEC hydrogel formation was, however, strongly influenced by the molar mass and oxidation degree of CMC, as well as by the weight ratio between CS and polyanion (Table 1). Thus, the PEC hydrogels prepared with CMC1 had low stability, their decomplexation occurring during washing or drying steps. Furthermore, PEC hydrogels were not obtained at 1/1 weight ratio between CS and DA-CMC1. This could also be related to the low molar mass of CMC1, which did not afford an extended chain entanglement following the protonation of the reaction mixture. From this point forward, these systems will not be discussed.

**Figure 5:**
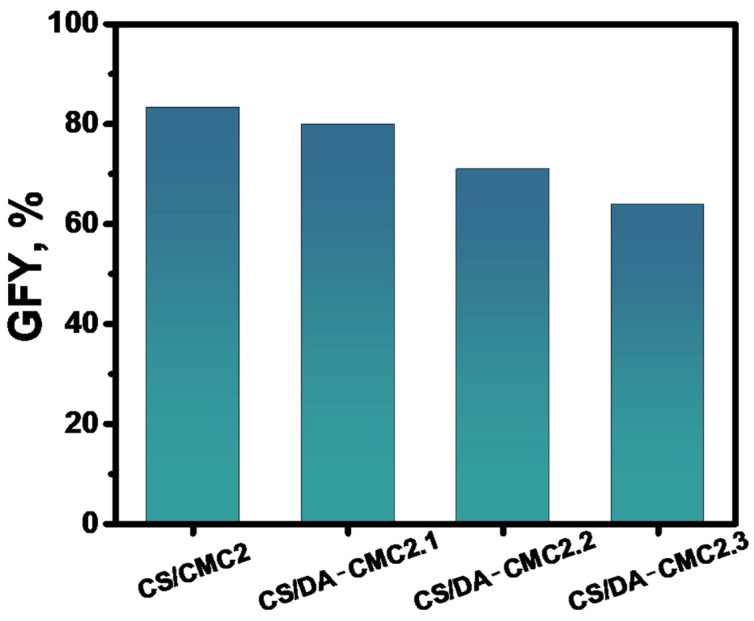
*GFY* of hydrogels prepared at 1/1 weight ratio between CS and CMC2 with different oxidation degrees.

When using CMC2, DA-CMC2.1, DA-CMC2.2 or DA-CMC2.3 as polyanions, more stable hydrogels were formed, irrespective of the weight ratio between components (Table 1). It is worth noting that all DA-CMC2 hydrogels were brittle after drying, a consequence of the formation of a dense covalent cross-linked network via imine bonds. The *GFY* of the hydrogels prepared at 1/1 weight ratio between CS and non-oxidized or oxidized CMC2 decreased from about 83% to about 64% as the oxidation degree of CMC2 increased (Figure 5). Experimentally, this was correlated with the increase in the hydrogels’ brittleness following the increase in imine bonds content, which induced an easier fracture development.

**Figure 6:**
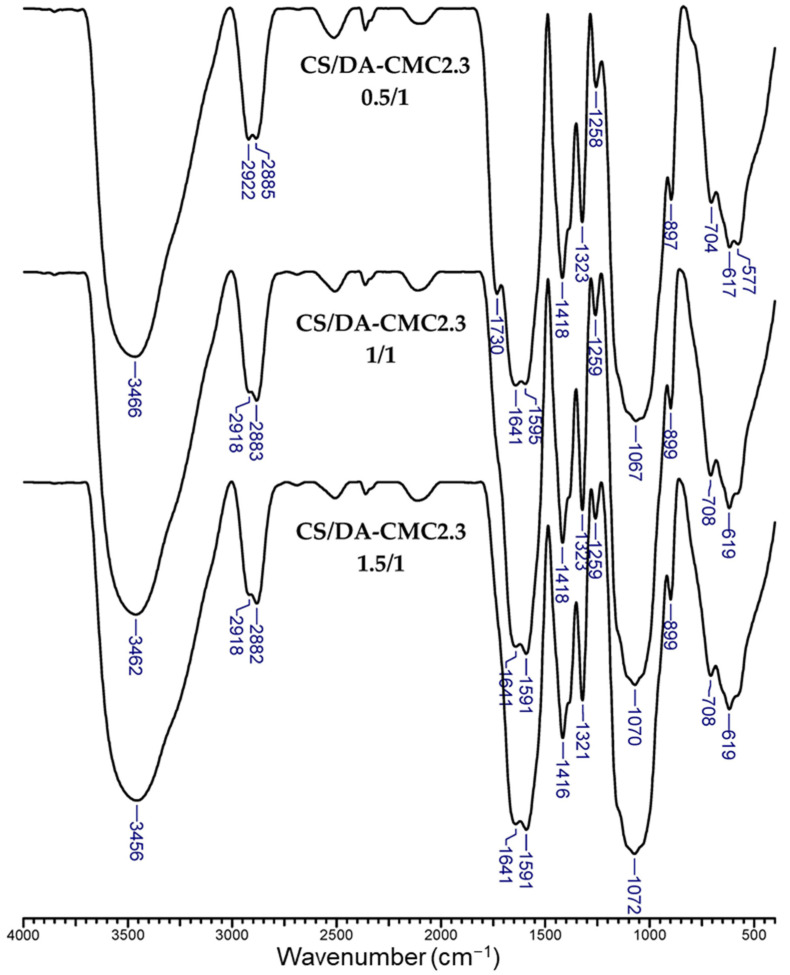
FTIR spectra of CS/DA-CMC2.3 PEC sponges as a function of the weight ratio between components.

FTIR spectra of CS/CMC2 and CS/DA-CMC2.3 hydrogels prepared at different weight ratios between components (0.5/1, 1/1 or 1.5/1) are presented in Figure S4 and Figure 6, respectively.

All spectra exhibited strong and broad bands at 3443–3466 cm^−1^, that are attributed to O-H and N-H bonds’ stretching vibrations, and to inter- and intramolecular hydrogen bonds from the constituent polysaccharides. The bands at 2918–2922 cm^−1^ and at 2855–2891 cm^−1^ are characteristic of the asymmetric and symmetric stretching vibrations of C-H groups, respectively. The bands assigned to aldehyde C=O groups (1738–1744 cm^−1^) were not visible in the FTIR spectra of CS/DA-CMC2.3 gels prepared at 1/1 and 1.5/1 weight ratios. Furthermore, the intensity of this band significantly diminished in the case of CS/DA-CMC2.3 gel prepared at 0.5/1 weight ratio. This supports the involvement of carbonyl groups in imine bonds formation. The bands at 1589–1595 cm^−1^ are characteristic of the vibrations of primary amine groups in CS [66]. The new bands appearing at 1321–1323 cm^−1^ in the spectra of CS/DA-CMC2.3 gels could be related to the deprotonation of carboxyl groups (-COOH → -COO¯) following their involvement in electrostatic interactions, and/or to the stretching vibrations of C-N bonds (amide III) in CS [48,62,66]. The bands situated at 802–804 cm^−1^ in the FTIR spectra of CMC2 and DA-CMC2.3, assigned to vicinal hydroxyl groups, also disappeared in the spectra of all gels, which is consistent with the involvement of hydroxyl groups in hydrogen bonds formation. To sum up, FTIR spectra demonstrate the intricacy of interactions that are involved in the stabilization of both types of gels.

**Figure 7:**
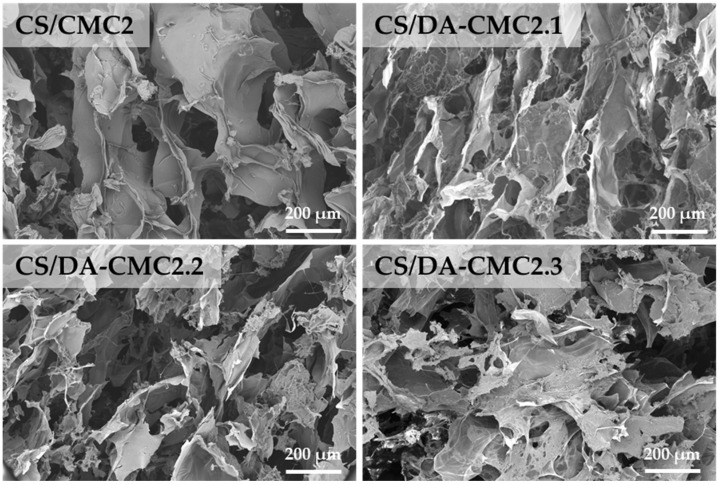
SEM micrographs of hydrogels prepared at 1/1 weight ratio between CS and CMC2 with different oxidation degrees.

The cross-sectional SEM micrographs showed that all hydrogels prepared at a 1/1 weight ratio between CS and CMC2 or DA-CMC2, with varying oxidation degrees, presented a porous microstructure, with interconnected pores (Figure 7). The highly macroporous structure of CS and CMC2 or DA-CMC2 hydrogels was also confirmed by the porosity values which ranged from 97.53% to 98.33%. These results are in accordance with those reported for other porous materials [67,68].

Such a sponge-like micromorphology is recognized as facilitating the mass transfer phenomenon; hence, the developed hydrogels could harness a great deal of interest as sorbents for the decontamination of aqueous media by various pollutants, including heavy metal ions, oxyanions, dyes, or pesticides [36,37,69,70]. However, it is evident that the average pore diameter of the hydrogels prepared using DA-CMC2 was lower than that of the CS/CMC2 sample, indicating the formation of more compact networks via covalent cross-linking (Figure 7). Thus, the average pore size decreased from 98 ± 29 μm (CS/CMC2 sample) to 58 ± 18 μm, 53 ± 12 μm and 47 ± 8 μm when DA-CMC2 with an *AC* content of 23.8% (CS/DA-CMC2.1 sample), 40.7% (CS/DA-CMC2.2 sample), and 60.2% (CS/DA-CMC2.3 sample), respectively, were used for the preparation of hydrogels.

**Figure 8:**
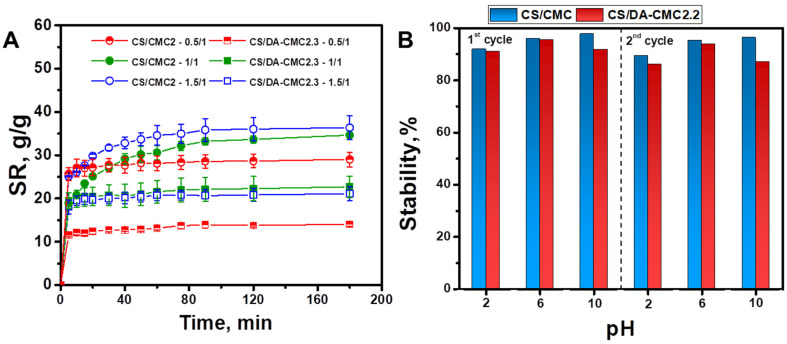
(**A**) Swelling kinetics of CS/CMC2 and CS/DA-CMC2.3 hydrogels with various compositions. (**B**) Stability of CS/CMC and CS/DA-CMC2.2 hydrogels in aqueous media with different pH values.

The swelling kinetics gives valuable information about the porosity and water diffusion within a hydrogel’s structure [34,71,72]. Figure 8A depicts the *SR* evolution as a function of time for the CS/CMC2 and CS/DA-CMC2.3 hydrogels prepared at different weight ratios between components.

As seen in Figure 8A, the CS/DA-CMC2 hydrogels exhibited lower equilibrium swelling capacities than the physically cross-linked CS/CMC2 ones. For both types of hydrogels, the lowest equilibrium swelling capacities were recorded for those prepared using an excess of CMC2 or DA-CMC2.3 (i.e., 0.5/1 CS/polyanion weight ratio). The equilibrium of swelling was reached very fast (less than 5 min) in the case of CS/DA-CMC2.3 hydrogels, while the CS/CMC2 hydrogels prepared at 0.5/1 and 1/1 weight ratios swelled steadily until equilibrium (approximately 90 min). The absorption of water by hydrogels is driven by capillary suction and by the increase in inner dielectric constant, until the osmotic pressure is counterbalanced by the elastic retraction force of the network [46,73]. The obtained swelling data support the idea that the covalent cross-linked CS/DA-CMC2.3 hydrogels are stiffer than the CS/CMC2 ones, and, thus, that the water absorption is driven mainly by capillary suction. The later hydrogels, stabilized by electrostatic interactions and hydrogen bonds, have a more elastic nature, with the absorption of water diminishing the interaction energy between the functional groups of CS (-NH_2_ and -OH) and CMC2 (-COOH and -OH).

**Figure 9:**
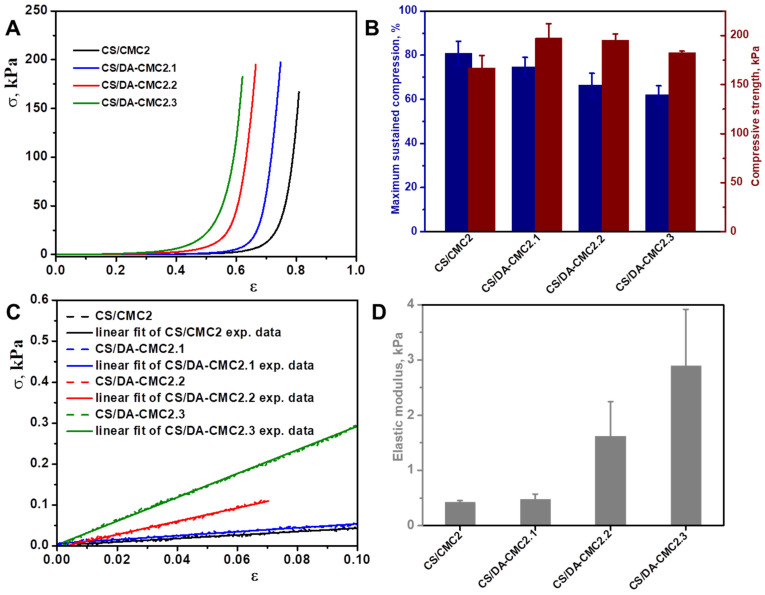
Mechanical properties of CS/CMC2 and CS/DA-CMC2 hydrogels: (**A**) Stress-strain curves; (**B**) Values of the maximum sustained compression (dark blue columns) and compressive nominal stress (wine columns); (**C**) The initial linear part in the stress-strain profiles used to calculate the elastic modulus; (**D**) Values of elastic modulus. All results are expressed as the average of at least three independent analyses ± SD.

The stability of CS/CMC and CS/DA-CMC2.2 hydrogels in aqueous media with different pH values was further investigated. Thus, PEC samples were kept first in pH 2, pH 6 or pH 10 for 3 h and then the erosion medium was removed, and the samples were freeze-dried and weighed again. As can be seen in Figure 8B, the CS/CMC and CS/DA-CMC2.2 hydrogels exhibited a remarkable stability in the investigated pH range, even after the second cycle of erosion. Secondly, we used the uniaxial compressive tests to assess the mechanical stability of the hydrogel matrices. The stress-strain curves, the elastic modulus, the maximum sustained compression, and compressive strength values of the CS/CMC, CS/DA-CMC2.1, CS/DA-CMC2.2, and CS/DA-CMC2.3 hydrogels in swollen states are presented in Figure 9. The stress–strain (σ-ε) profiles depicted in Figure 9A for the PEC samples reveal typical elastic performance that is characteristic of macroporous materials. The CS/CMC, CS/DA-CMC2.1, CS/DA-CMC2.2, and CS/DA-CMC2.3 hydrogels can sustain compression values of 80.88%, 74.76%, 66.49%, and 62.05%, respectively, without any crack development in the gel network (Figure 9A).

It should be emphasized that when using DA-CMC2 in the preparation of the hydrogels, the compressive strength slightly increased, while the maximum sustained compression decreased, which indicate an increase in the network rigidity due to the covalent cross-linking of the matrix (Figure 9B,D). For instance, the CS/DA-CMC2.1 and CS/DA-CMC2.3 hydrogels sustained 74.76% and 62.05% compression, respectively, at a compressive nominal stress of 197.43 kPa and 182.51 kPa, respectively, while the CS/CMC2 hydrogels sustained 80.88% compression at a compressive nominal stress of 166.987 kPa. The elastic moduli of the chemically cross-linked hydrogels, calculated as the slope of the initial linear portion in the stress-strain profiles (Figure 9C), increased with the increase in the *AC* content within DA-CMC2 (Figure 9D), indicating a denser network for these materials. The improvement of the mechanical features of the chemically cross-linked hydrogels, in comparison to those physically cross-linked, could be attributed to the decrease in pore sizes (Figure 7), and reduced *SR* values (Figure 8A). These results are in agreement with the data reported on CS/oxidized starch PEC sponges [47], CS/dextrin sponges [74], CS/dextran composites [75], and CS/agarose blends [76].

### 3.3. Sorption of Fungicide Formulations

**Figure 10:**
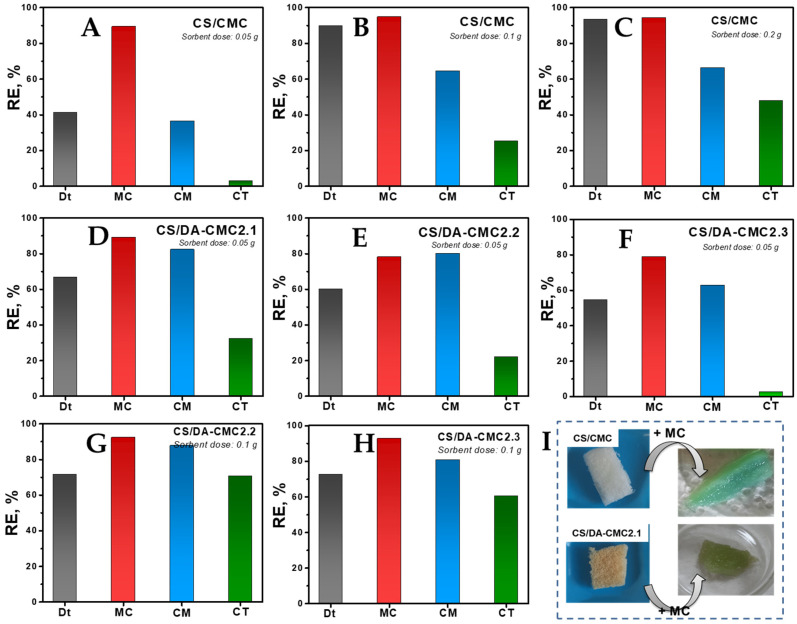
Effect of sorbent type and dose on *RE* of fungicides (**A**–**H**). Optical images of CS/CMC and CS/DA-CMC2.1 hydrogels before and after sorption of MC (**I**).

The fungicide formulations used in this work were commercial grade. Dt and CM contain, as the active ingredient, mancozeb, a manganese/zinc ethylene bis-dithiocarbamate, in proportions of 80% and 18–20%, respectively. MC and CM contain 40.6% and 50%, respectively, of copper oxychloride (Cu_2_(OH)_3_Cl). MC also contains about 8.4% iprovalicarb, while CT contains pyraclostrobin and metiram in proportions of 5% and 55%, respectively. CM also contains 5% cymoxanil. In addition to the active substances (Figure 2), all fungicide formulations also contain significant amounts of undisclosed inert components, added to improve their ease of application, the self-life, and/or to prevent them from degradation. Figure 10A–H depicts the effect of sorbent dose on the *RE* of fungicides using CS/CMC, CS/DA-CMC2.1, CS/DA-CMC2.2 and CS/DA-CMC2.3 hydrogels prepared at 1/1 weight ratio between components.

At a sorbent dose of 0.05 g, the *RE* of fungicides by the CS/CMC hydrogels followed the order MC (89%) > Dt (41%) > CM (36%) > CT (3%) (Figure 10A). The fungicides’ *RE* by the CS/CMC hydrogels increased significantly at higher sorbent doses (Figure 10B,C), reaching 94%, 93%, 66% and 48% for MC, Dt, CM and CT, respectively, at 0.2 g sorbent dose. The hydrogels prepared using DA-CMC2 (i.e., CS/DA-CMC2.1, CS/DA-CMC2.2 and CS/DA-CMC2.3) showed improved *RE* for CM and CT (Figure 10D–H) compared to the CS/CMC hydrogels, irrespective of the sorbent dose used. However, the increase in CMC oxidation degree had a detrimental effect on the *RE* of fungicides at constant sorbent dose. The highest *RE* values for CM (88%) and CT (71%) were obtained using the CS/DA-CMC2.2 hydrogels at 0.1 g sorbent dose (Figure 10G).

All fungicide formulations were negatively charged in aqueous media, as illustrated by the ζ values presented in Figure 2. Therefore, a probable interaction mechanism for the sorption of fungicides by the developed PEC hydrogels could be based on electrostatic attractions with the amino groups of CS. Taking into account the chemical structures of active ingredients in the fungicide formulations, other types of interactions, such as hydrogen bonds (with -OH and/or -COOH groups on the sorbents) or hydrophobic interactions are also possible [77,78]. In addition to the chemical composition of the PEC hydrogels, the type and content of heavy metal ions in the active ingredients of fungicide suspensions could also play important roles. As seen above, higher *RE* values have been recorded for the fungicides that contain Cu_2_(OH)_3_Cl. The preferential adsorption of Cu(II) onto polysaccharide-based hydrogels has been thoroughly demonstrated in the last years, largely because Cu(II) ions easily form four and five coordination complexes, having a distorted Jahn–Teller structure with the ligand groups on the polysaccharides, while the complexes with Zn(II) and Mn(II) ions adopt an octahedral spatial configuration [79,80]. Figure 2 also shows that the median diameters (D50) of particles in the suspensions of fungicides were 1.99 µm, 2.26 µm, 2.44 µm and 2.61 µm for CM, CT, MC, and Dt, respectively. Taking into account the variation in pore sizes within the PEC hydrogels (Figure 7), one would also expect a significant contribution of the intraparticle diffusion mechanism on the sorption of fungicides, which would increase with the increase in oxidation degree of DA-CMC2.

**Figure 11:**
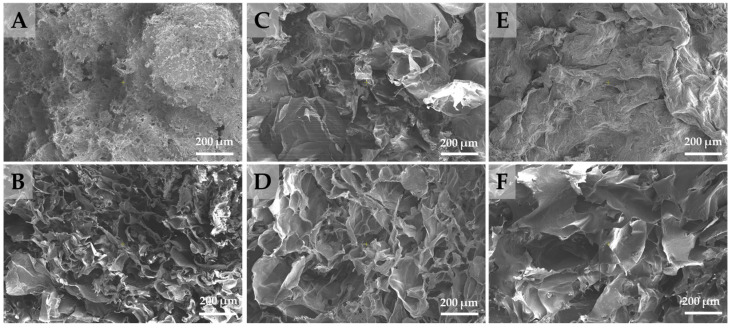
SEM micrographs of CS/CMC2 (**A**,**C**,**E**) and CS/DA-CMC2.1 (**B**,**D**,**F**) hydrogels after the sorption of Dt (**A**,**B**), MC (**C**,**D**) and CM (**E**,**F**).

The SEM micrographs of CS/CMC and CS/DA-CMC2.1 hydrogels after the sorption of Dt, MC and CM (Figure 11) revealed a strong collapse of the polymeric networks, and a strong decrease in pore sizes. This indicates a strong interaction between the fungicides and the PEC hydrogels.

**Figure 12:**
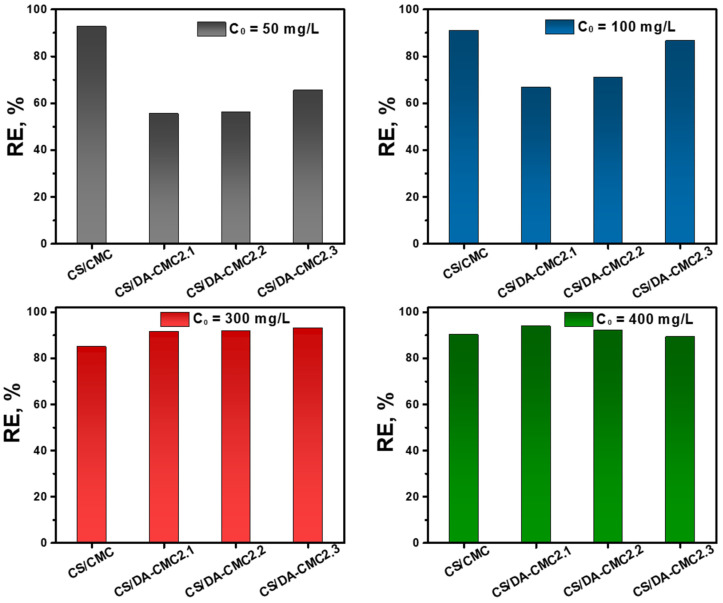
Effect of initial concentration on *RE* of MC by CS/CMC2, CS/DA-CMC2.1, CS/DA-CMC2.2 and CS/DA-CMC2.3 hydrogels.

To investigate the liquid–solid partition equilibrium, MC sorption by the PEC hydrogels at different initial suspension concentrations has been performed (Figure 12).

The sorption of fungicides by the developed hydrogels can be influenced by a complex set of parameters, including the hydrogel/fungicide affinity and the density of interactions. In Figure 12, the sorption of MC as a function of hydrogel type and the initial fungicide concentration is depicted. MC has a strongly negative zeta potential, as seen in Figure 2. Thus, it can easily interact with the CS/CMC2 hydrogels, even at lower initial concentrations, by electrostatic interactions with the amino groups of CS. In the case of the hydrogels prepared with DA-CMC2, the amino groups of CS were involved in Schiff base formation; hence, they could not form electrostatic interactions with the fungicide particles. The low *RE* values obtained for MC particles’ sorption by these hydrogels could be related to the lower hydrogel/fungicide density of interactions at low initial MC concentrations (i.e., 50 mg/L and 100 mg/L). On the other hand, the increase in MC concentration (300 mg/L and 400 mg/L) led to a higher density of interactions with the hydrogel surface. This favored the better adsorption of MC particles, hence higher RE values.

Our results on the sorption of fungicide formulations indicate that the hydrogels prepared using DA-CMC2 are more efficient sorbents for MC from aqueous media than CS/CMC2 ones.

## 4. Conclusions

In this work, new, porous, self-assembled hydrogels have been prepared using CS and CMC or DA-CMC, by combining the delayed protonation of reaction mixture under acetic acid atmosphere with a freeze-drying technique and tested as sorbents for different commercial fungicide formulations. Firstly, DA-CMC polymers with different oxidation degrees have been synthesized, starting from CMC with two different molar masses, by varying the AGU:NaIO_4_ molar ratio. Then, CS/CMC hydrogels stabilized by electrostatic interaction and/or hydrogen bonds, and CS/DA-CMC hydrogels stabilized by in situ imine bonds formation, have been prepared. It was found that the formation of PEC hydrogels was significantly influenced by the molar mass and oxidation degree of CMC, as well as by the weight ratio between the CS and polyanions. Thus, the hydrogels prepared with CMC1 had low stability, with their decomplexation occurring during washing or drying steps. Furthermore, PEC hydrogels have not been obtained at 1/1 weight ratio between CS and DA-CMC1, which was attributed to the low molar mass of CMC1. When CMC2, DA-CMC2.1, DA-CMC2.2 or DA-CMC2.3 were used as polyanions, more stable PEC hydrogels were obtained, irrespective of the weight ratio between the components. The FTIR analysis proved the formation of PEC hydrogels. The cross-sectional SEM micrographs showed that all PEC hydrogels exhibited a porous microstructure, with interconnected pores, whose sizes depended on the polyanion structure. It was shown that the pore sizes of the PEC hydrogels prepared using DA-CMC2 were smaller than those of the CS/CMC hydrogels, indicating the formation of more compact networks via covalent cross-linking in the former. The equilibrium swelling study indicated lower swelling ratio values for chemically cross-linked CS/DA-CMC2 hydrogels than for the physically cross-linked CS/CMC2 ones, as expected.

The PEC hydrogels were tested as sorbents for different commercial fungicide formulations. The hydrogels prepared using DA-CMC2 showed improved *RE* for CM and CT compared to the CS/CMC hydrogels, irrespective of the sorbent dose used. However, the increase in CMC oxidation degree had a detrimental effect on the *RE* of fungicides at constant sorbent dose. The highest *RE* values for CM (88%) and CT (71%) were obtained using the CS/DA-CMC2.2 hydrogels at 0.1 g sorbent dose. This indicates that the hydrogels prepared using DA-CMC2 are more efficient sorbents for MC from aqueous media than CS/CMC2 ones.

## Figures and Tables

**Table 1:** Preparation conditions of CS/CMC and CS/DA-CMC PEC sponges.

PEC	m_CS_, g	C_polyanion_, %	m_polyanion_, g	Notes
CS/CMC1–0.5/1	0.05	1	0.1	Partial decomplexation during washing
CS/CMC1–1/1	0.1	1	0.1	Partial decomplexation during drying
CS/CMC1–1.5/1	0.15	1	0.1	Partial decomplexation during washing
CS/DA-CMC1	0.1	1	0.1	Does not form gel
CS/CMC2–0.5/1	0.1	2	0.2	Gel stable during washing
CS/CMC2–1/1	0.4	2	0.4	Gel stable during washing
CS/CMC2–1.5/1	0.3	2	0.2	Gel stable during washing
CS/DA-CMC2.1–1.1	0.24	2	0.24	Gel stable during washing; Brittle after drying
CS/DA-CMC2.2–1.1	0.24	2	0.24	Gel stable during washing; Brittle after drying
CS/DA-CMC2.3–0.5/1	0.2	2	0.4	Gel stable during washing; Brittle after drying
CS/DA-CMC2.3–1/1	0.4	2	0.4	Gel stable during washing; Brittle after drying
CS/DA-CMC2.3–1.5/1	0.3	2	0.2	Gel stable during washing; Brittle after drying

**Table 2:** Synthesis conditions and main characteristics of DA-CMC.

Sample	CMC, mmol	NaIO_4_, mmol	*AC*, %	*C_COOH_*, mmol/g
CMC1	-	-	-	3.36
DA-CMC1	22.90	22.90	68.9	3.78
CMC2	-	-	-	3.72
DA-CMC2.1	21.37	3.562	23.8	3.77
DA-CMC2.2	21.37	8.905	40.7	3.84
DA-CMC2.3	21.37	21.37	60.2	4.31

## Data Availability

Not applicable.

## Supplementary Materials

The following supporting information can be downloaded at: https://www.mdpi.com/article/10.3390/polym15173496/s1, Figure S1. Conductometric titration profiles of the non-oxidized and oxidized CMC1 and CMC2. Figure S2. FTIR spectra of DA-CMC2.2 and DA-CMC2.3. Figure S3. Optical images of CS/CMC2 and CS/DA-CMC2.3 hydrogels prepared at 1:1 wt. ratio. Figure S4. FTIR spectra of CS/CMC2 PEC sponges prepared at different weight ratios. Table S1. NH_2_/COOH and NH_2_/C=O molar ratio as a function of hydrogels composition.
