# Supplementary material for: Self-Assembled Chitosan/Dialdehyde Carboxymethyl Cellulose Hydrogels: Preparation and Application in the Removal of Complex Fungicide Formulations from Aqueous Media
Source: Polymers (Basel). 2023 Aug 22;15(17):3496. doi: 10.3390/polym15173496 (PMC10490195; doi:10.3390/polym15173496)
Supplement: Supplementary file 1 [file polymers-15-03496-s001.zip › polymers-2503242-supplementary.pdf]

SUPPLEMENTARY MATERIAL

Self-Assembled Chitosan/Dialdehyde Carboxymethyl Cellulose Hydrogels: Preparation and Application in the Removal of Complex Fungicide Formulations from Aqueous Media

Claudiu-Augustin Ghiorghita, Maria Marinela Lazar, Luminita Ghimici and Maria Valentina Dinu *

"Petru Poni" Institute of Macromolecular Chemistry, Grigore Ghica Voda Alley 41 A, 700487 Iasi, Romania; claudiu.ghiorghita@icmpp.ro (C.-A.G.); maria.lazar@icmpp.ro (M.M.L.); lghimici@icmpp.ro (L.G.)

* Correspondence: vdinu@icmpp.ro

Figure S1. Conductometric titration profiles of the non-oxidized and oxidized CMC1 and CMC2.

Figure S2. FTIR spectra of DA-CMC2.2 and DA-CMC2.3.

Figure S3. Optical images of CS/CMC2 and CS/DA-CMC2.3 hydrogels prepared at 1:1 wt. ratio.

Figure S4. FTIR spectra of CS/CMC2 PEC sponges prepared at different weight ratios.

Table S1. NH_2/COOH and NH2/C=O molar ratio as a function of hydrogels composition.

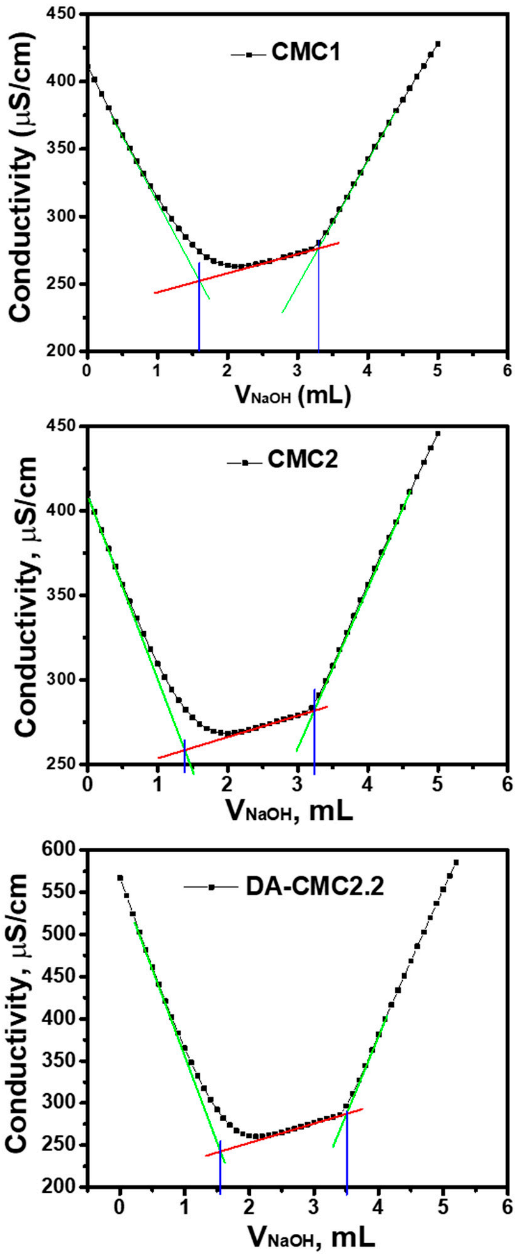

Figure S1. Conductometric titration profiles of the non-oxidized and oxidized CMC1 and CMC2.

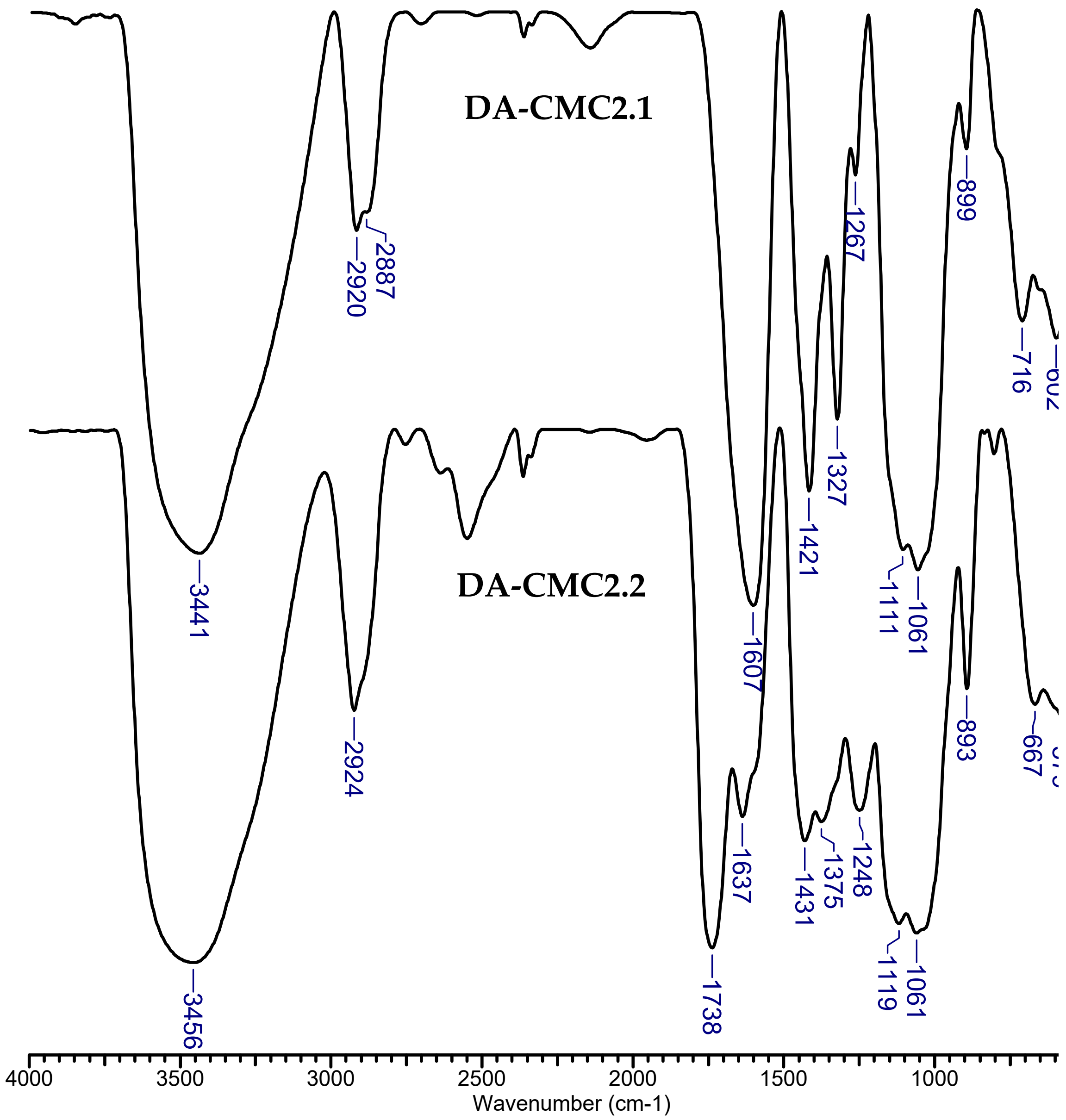

Figure S2. FTIR spectra of DA-CMC2.2 and DA-CMC2.3.

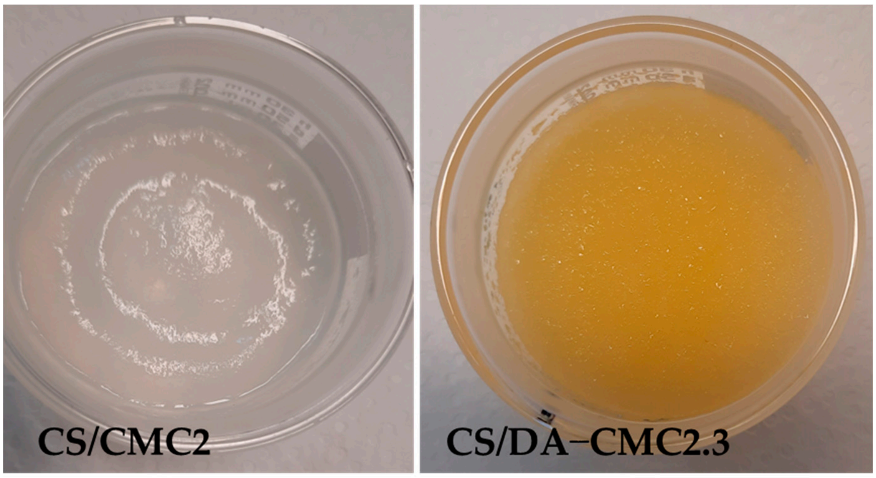

Figure S3. Optical images of CS/CMC2 and CS/DA-CMC2.3 hydrogels prepared at 1:1 wt. ratio.

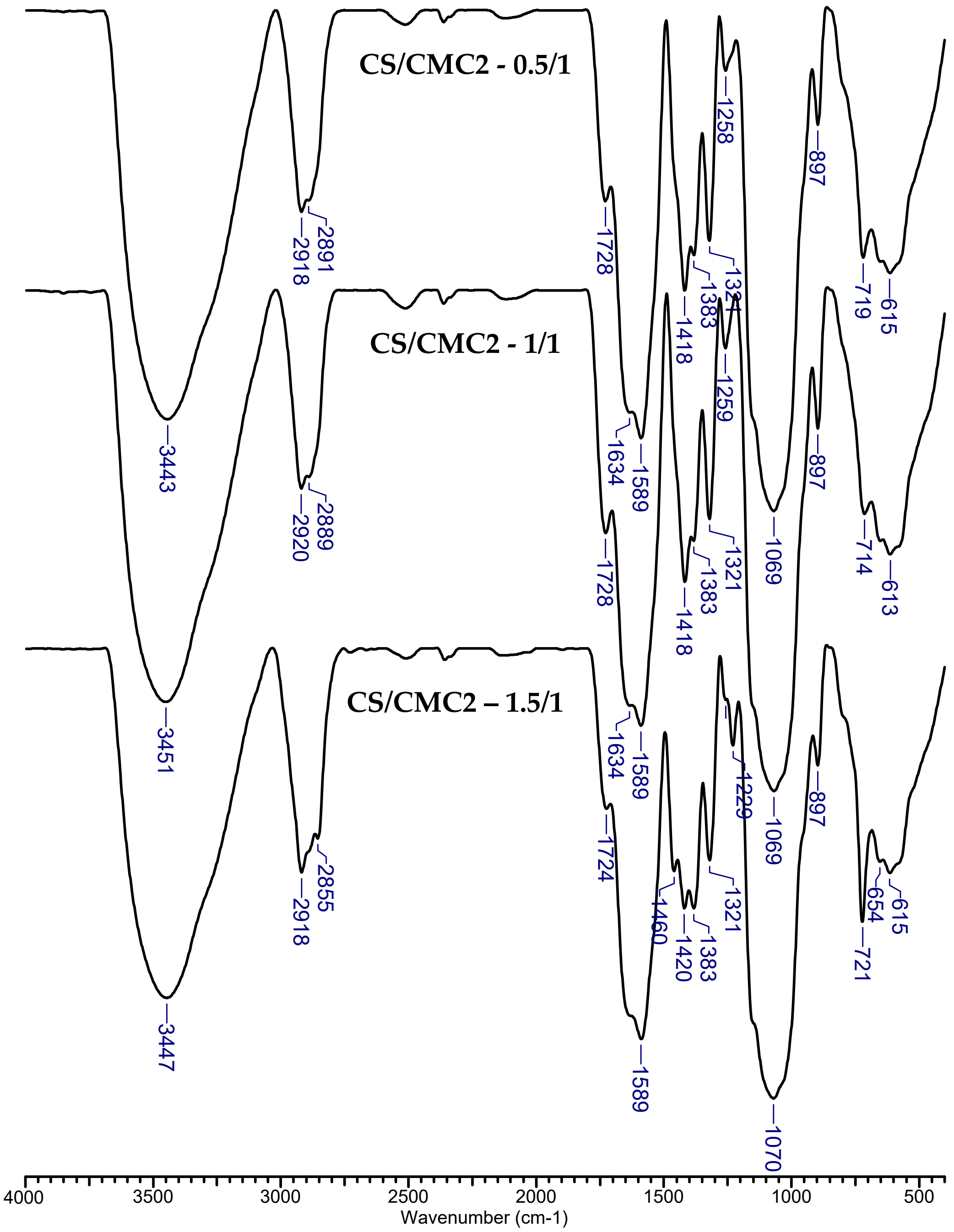

Figure S4. FTIR spectra of CS/CMC2 PEC sponges prepared at different weight ratios.

Table S1. NH_2/COOH and NH_2/C=O molar ratio as a function of hydrogels composition.

PEC	Molar ratio	
	NH_2/COOH	NH_2/C=O
**CS/CMC1 – 0.5/1**	0.65	-
**CS/CMC1 – 1/1**	1.30	-
**CS/CMC1 – 1.5/1**	1.95	-
**CS/DA-CMC1**	1.16	0.78
**CS/CMC2 – 0.5/1**	0.59	-
**CS/CMC2 – 1/1**	1.18	-
**CS/CMC2 – 1.5/1**	1.76	-
**CS/DA-CMC2.1 – 1.1**	1.16	0.25
**CS/DA-CMC2.2 – 1.1**	1.14	0.44
**CS/DA-CMC2.3 – 0.5/1**	0.51	1.29
**CS/DA-CMC2.3 – 1/1**	1.01	0.65
**CS/DA-CMC2.3 – 1.5/1**	1.52	0.43
